# Synergistic governance: China's roadmap to improved health through climate and clean air actions

**DOI:** 10.1016/j.ese.2024.100447

**Published:** 2024-07-04

**Authors:** Tao Xue, Tong Zhu

**Affiliations:** Institute of Reproductive and Child Health, National Health Commission Key Laboratory of Reproductive Health and Department of Epidemiology and Biostatistics, Ministry of Education Key Laboratory of Epidemiology of Major Diseases (PKU), School of Public Health, Peking University Health Science Centre, Beijing, China; SKL-ESPC and SEPKL-AERM, College of Environmental Sciences and Engineering, Center for Environment and Health, Peking University, Beijing, 100871, China

## Abstract

• Health benefits from China's pollution-carbon co-control actions have already been seen.
• Co-control of air pollution and greenhouse gases can avoid premature deaths.
• More comparative evaluations of the health impacts of specific policies are needed.

The COP28's inaugural Health Day highlighted the growing recognition of the connection between climate and health, while China's State Council released its third air quality action plan aiming at ongoing health improvement. The Synergetic Roadmap project [1], which aims to track China's progress towards carbon neutrality and clean air, provides valuable insights to answer a set of intriguing questions concerning environmental health determinants, their source sectors, and the methods for harmonizing disparate actions to maximize health gain.

The Synergetic Roadmap project offers a wellspring of knowledge to address these queries. The 2022 report, *accelerating transition in key sectors*, not only shows pathways connecting health with climate and air quality, but also underscores the attributable burden of premature deaths [1]. The climatic factors including heatwaves, heavy rainfall, and floods are shown to amplify the risk of infectious and non-communicable diseases, while air pollutants including fine particulate matter (PM_2.5_), ozone (O_3_), and nitrogen dioxides (NO_2_) contribute to premature mortalities by the millions per year [2,3]. A groundbreaking aspect of Lei et al. (2024) [1] is its endeavor to trace pollution exposure and health impacts systematically, incorporating indicators of human health impacts in the assessment system, and aptly conveying the health benefits from environmental interventions to the policymakers and the public in China. However, it is worth to highlight the health impacts of air pollution from multiple perspectives, which should be considered by the report in future. For instance, PM_2.5_ exposure has been linked to not only physical illnesses but also mental disorders [4].

The comparative analyses in Lei et al. (2024) [1] suggested air pollution as a more considerable determinant of health than extreme weather events, mainly due to its widespread and enduring nature, and further suggested long-term PM_2.5_ exposure as the dominant contributor to the burden of diseases. The authors emphasize that significant reductions in carbon and air pollutant emissions and great health improvement will be achieved by optimizing the choice of low-carbon materials or services in energy, production and consumption. The finding fortifies the third action plan's strategy, which targets a sustained reduction in PM_2.5_ levels. Ongoing work of the Synergetic Roadmap project might serve as a critical reference for framing future climate or clean air actions, by ranking different environmental determinants by their health effects.

Moreover, Lei et al. (2024) [1] maps out how climate actions and clean air actions can be cohesively managed to co-control greenhouse gases and air pollutants, thus optimizing health outcomes. Lei and colleagues aim to monitor the progress in these co-controls through a detailed evaluation using 20 different indicators. The 2022 report [1] highlights important milestones in key sectors that can help speed up co-controls, such as reducing coal use in power generation, slowing down the growth of steel and cement production, and encouraging the use of electric vehicles. The report also notes that some health benefits from these milestones have already been seen in China, such as the reduction of premature deaths from switching to cleaner energy sources in the power sector. Nonetheless, besides solutions to air pollution, there is a need for thorough and comparative evaluations of the health impacts of specific synergistic policies (e.g., black carbon emission controls) in future reports, in order to develop a health-focused decision-making framework that integrates climate action and clean air initiatives. For instance, since recent findings suggest the co-exposure to PM_2.5_ and O_3_ episodes can be more hazardous than the single-pollutant exposure [5], the co-control strategies help to maximize health benefits. In addition, unbalanced synergistic downward trends in pollutants [6] and carbon among provinces and cities may cause air pollution and climate change exposure disparities [7]. For instance, the temporal correlation between PM_2.5_ and O_3_ was positive in South China, but positive in North [6], which contributed to the geographic inequality in air-pollution-associated premature deaths [2].

Endorsed by over 120 countries through the COP28 Declaration on Climate and Health, the commitment to improving health through environmental actions is gaining momentum. The Synergetic Roadmap project's efforts to develop and to track co-control strategies contribute to placing China at the forefront of this movement. The potential for a synergistic environmental governance approach will yield continuous health advancements [8].
